# Successful treatment of late obstetric ethylene glycol intoxication with ethanol via the enteral route: a case report

**DOI:** 10.3325/cmj.2023.64.436

**Published:** 2023-12

**Authors:** Dinka Lulić, Ivan Gornik, Jadranka Pavičić Šarić, Mirjana Mirić, Anita Lukić, Ileana Lulić

**Affiliations:** 1 Department of Emergency Medicine, University Hospital Center Zagreb, Zagreb, Croatia; 2 School of Medicine, University of Zagreb, Zagreb, Croatia; 3 Department of Anesthesiology, Intensive Care and Pain Medicine, University Hospital Merkur, Zagreb, Croatia; 4 Department of Anesthesiology, Reanimatology, Intensive Care and Pain Medicine, University Hospital Center Zagreb, Zagreb, Croatia; 5 Department of Anesthesiology, Reanimatology and Intensive Care Medicine, Varaždin General Hospital, Varaždin, Croatia

## Abstract

Late obstetric ethylene glycol intoxication represents a diagnostic challenge for acute care physicians and an impending life threat with life-long implications for both the mother and the fetus. The metabolism of ethylene glycol to its toxic metabolites during late pregnancy is unpredictable due to maternal physiological changes. Namely, the hallmark signs and symptoms of ethylene glycol intoxication can mimic those of late pregnancy-related high blood pressure disorders, which makes it difficult to correctly diagnose the condition. Therefore, it is crucial to promptly recognize late obstetric ethylene glycol intoxication and initiate specific treatment, but evidence-based recommendations are not available to guide its most effective emergent treatment. We present our department's emergent management of late-obstetric ethylene glycol intoxication. The parturient was stabilized by inhibiting ethylene glycol metabolism, alongside general supportive care measures. The enhancement of its toxic metabolites was eliminated by administering ethanol via the enteral route, which progressively improved the parturient’s clinical course and led to the on-term delivery of a healthy child. Our case shows the importance of a meticulous emergent assessment, prompt diagnosis, and carefully planned multidisciplinary treatment in the emergency department in improving outcomes after ethylene glycol intoxication in late pregnancy.

Late-obstetric ethylene glycol intoxication is a rare, life-threatening event (1). Hallmark signs and symptoms can mimic those of late pregnancy-related high blood pressure disorders (2). Thus, ethylene glycol intoxication during late pregnancy needs to be recognized early so that specific treatment modalities can be initiated (3). To date, no observational studies or randomized controlled trials have directly compared the effect of different late obstetric ethylene glycol intoxication treatment modalities on parturient outcomes (4). We present the first case of late obstetric ethylene glycol intoxication successfully treated with ethanol via the enteral route as an escape therapeutic intervention in the emergency department. As a result of the treatment, the parturient’s clinical course progressively improved, and she delivered a healthy child on-term.

## Case report

In March 2021, a 35-year-old woman, at 32 weeks gestation of her second pregnancy, was admitted to the emergency department, approximately eight hours after ingesting 500 mL of an anti-freeze product. The medical history revealed gestational diabetes during the first pregnancy, and a previous suicide attempt by ingesting benzodiazepines. On admission, the patient was somnolent, with a Glasgow Coma Score of 11 (E2, V4, M5), isochoric pupils sluggishly reacting to light, and unremarkable brainstem reflexes. She demonstrated the Kussmaul breathing pattern with normal oxygen saturation on room air, hemodynamic instability, and lowered body temperature.

Arterial blood gas analysis showed high anion gap metabolic acidosis, a significant osmolality gap, and elevated lactates. The complete blood count and liver function tests were within the reference ranges. However, calcium oxalate and glycolate crystals were present in the urine sediment. Toxicology tests were negative for all the toxins and drugs, except for elevated levels of benzodiazepines in the serum. The findings of point-of-care trans-abdominal ultrasound used for a rapid assessment of fetal viability were unremarkable.

At the time, high-performance liquid chromatography for detecting oxalate and glycolate in urine, and gas chromatography for detecting plasma ethylene glycol concentration were not available in the emergency department.

The parturient underwent prompt emergent stabilization, which consisted of general supportive care and administration of intravenous fluids and isotonic sodium bicarbonate. In parallel, a consultation with the Poison Control Centre was initiated. Since intravenous ethanol and fomepizole were not available, the parturient was immediately administered 40 mL of 40% ethanol via a nasogastric tube, followed by a continuous administration of 40% enteral ethanol, as we aimed to reach a blood ethanol level of about 1‰.

**Figure 1:**
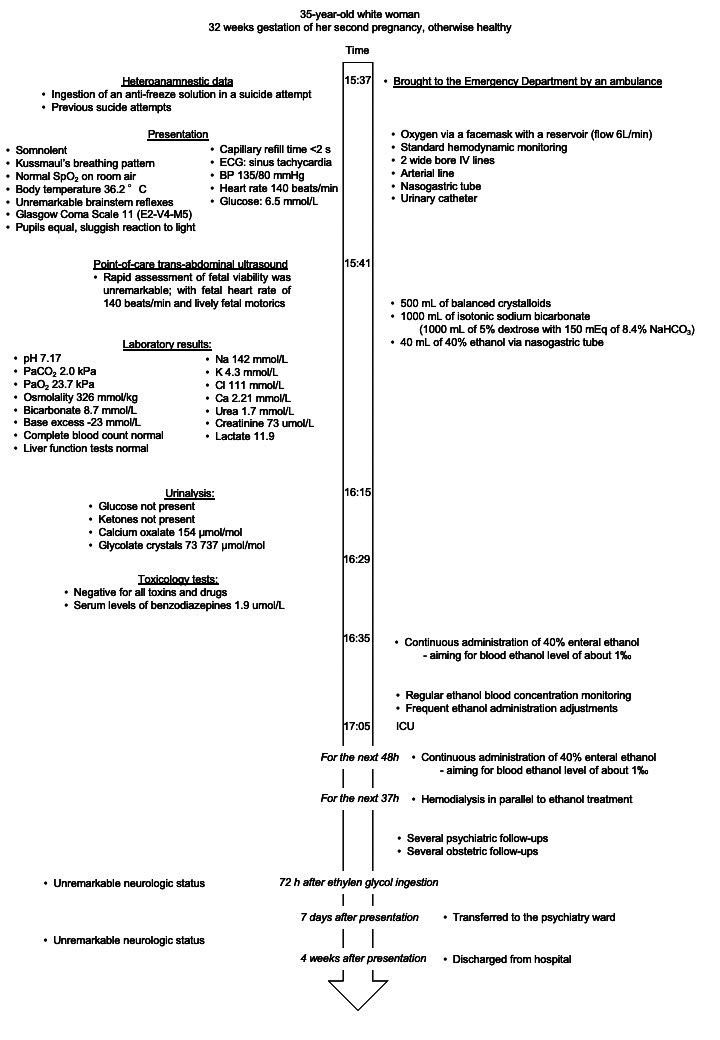
Timeline of diagnoses, laboratory and imaging results, and treatments of a 35-year-old woman, at 32 weeks gestation, after intoxication with ethylene glycol. SpO_2_ – peripheral saturation with oxygen, BP – blood pressure, IV – intravenously, ECG – electrocardiogram, PaO_2_ – partial pressure of oxygen in arterial blood, PaCO_2_ – partial pressure of carbon dioxide in arterial blood, E-V-M – eyes – voice – motoric, ICU – intensive care unit.

After the transfer to the intensive care unit (ICU), the treatment continued to optimize the parturient’s oxygenation and acid-base status. Upon admission to the ICU, a dialysis catheter was placed into the right subclavian vein. Along with the 40% ethanol administration via a nasogastric tube, continuous hemodialysis was continued for 37 hours. The parturient’s physical and neurologic status normalized 72 hours after the intoxication. During her stay in the ICU, regular psychiatric and obstetric examinations were performed. Seven days after admission, the parturient was transferred to the psychiatric ward. Four weeks after the admission, she completely clinically recovered and was discharged from hospital. The timeline of diagnoses, laboratory and imaging results, and treatments is depicted in Figure 1.

In the following months, the parturient and her family members adhered to the given recommendations. Four weeks after discharge, the parturient gave birth to a healthy child without an apparent neurological deficit.

## Discussion

The parturient, who had high anion gap metabolic acidosis and a significant osmolality gap following ethylene glycol intoxication, was successfully treated with ethanol administration via the enteral route in the emergency department, along with general supportive measures.

Late obstetric ethylene glycol intoxication may produce irreversible organ dysfunction if treated later in its course and is associated with high morbidity and mortality of both the mother and the fetus. However, most of such intoxications present a diagnostic challenge for critical care physicians due to the presence of diverse clinical signs and symptoms combined with several possible differential diagnoses related to pregnancy. In addition, most emergency departments have limited on-site abilities for the quantitative analysis of ethylene glycol concentrations. In such situations, the main diagnostic clue for obstetric ethylene glycol intoxication is a combination of profound anion gap metabolic acidosis, elevated osmolality gap, and the presence of envelope-shaped and needle-shaped oxalate crystals in the urine, coupled with patients' clinical inebriation, altered mental status, and acute renal failure (5).

The treatment focuses on the inhibition of ethylene glycol metabolism and elimination enhancement of the unmetabolized parent compound and its toxic metabolites, accompanied by an early antidote administration (6). Current guidelines recommend the use of fomepizole as a first-line treatment after ethylene glycol ingestion in all stages of pregnancy. However, the decision to use ethanol or fomepizole depends on the availability and costs of the antidote, parturient characteristics, and critical care physicians' experience with a specific antidote (7,8). Since ethylene glycol and its metabolites are dialyzable poisons due to their small size, high water solubility, absence of protein binding, and low volume of distribution, additional critical component in the management of ethylene glycol intoxication is the institution of hemodialysis protocols (4).

In our emergency department, fomepizole was not available, nor could it have been provided in a reasonable time. Instead, administration of ethanol via the enteral route was used as an escape therapeutic intervention, which progressively improved our parturient’s clinical course. Furthermore, concurrent 40% ethanol administration via a nasogastric tube, along with continuous hemodialysis, was extended for 37 hours in the ICU to substantially increase the total ethylene glycol clearance.

To our knowledge, this is the first case describing the clinical management of obstetric ethylene glycol intoxication during the third semester of pregnancy with successful outcomes. Kralova et al (2) reported on a case of obstetric ethylene glycol intoxication during the second trimester of pregnancy. In their patient, an emergent Cesarean section had to be undertaken after treatment with ethanol via enteral route and hemodialysis administration as the health status of the parturient was substantially impaired (2). This underpins the already reported toxicity of ethanol during pregnancy, which may result in preterm birth (9). On the other hand, it may be theoretically safer to treat pregnant patients with fomepizole, as described by Harbon et al in their case of successful clinical management of obstetric ethylene glycol intoxication during the first trimester of pregnancy (10).

Our case demonstrates the importance of early administration of ethanol via the enteral route for obstetric ethylene glycol intoxication in an emergency setting as an escape therapeutic intervention. Further, a meticulous emergent assessment, expeditious diagnosis, and carefully planned multidisciplinary treatment in the emergency department have proven to be vital for improving outcomes after ethylene glycol intoxication in late pregnancy.
